# Implementing an eHealth Model of Care for Pediatric Patients and Families at the End of Treatment for Acute Lymphoblastic Leukemia (EMERGE): Type 2 Nonrandomized Hybrid Implementation-Effectiveness Trial Study Protocol

**DOI:** 10.2196/85901

**Published:** 2026-02-26

**Authors:** Maria C McCarthy, Chris Williams, Michelle Tennant, Richard De Abreu Lourenco, Hannah Pring, Katie Moore, Jane Templeton, Ken Knight, Peter Downie, Stephen Hearps, Cinzia De Luca

**Affiliations:** 1 Clinical Sciences, Murdoch Children's Research Institute, 50 Flemington Road, Parkville, 3052, Australia, 61 93456866; 2 Department of Paediatrics, The University of Melbourne, Parkville, Australia; 3 Children's Cancer Centre, Royal Children's Hospital, Parkville, Australia; 4 Victorian Paediatric Integrated Cancer Service, Parkville, Australia; 5 Centre for Health Economics Research and Evaluation, University of Technology, Sydney, Australia; 6 Children's Cancer Centre, Monash Health, Clayton, Australia; 7 Department of Paediatrics, Monash University, Clayton, Australia; 8 Department of Critical Care, University of Melbourne, Parkville, Australia

**Keywords:** childhood cancer, end-of-treatment, model of care, intervention, telehealth, e-health

## Abstract

**Background:**

Despite increasing survival rates for childhood cancers, physical and psychological late effects are common. The end-of-treatment period is recognized as a complex transition period, and there are few evidence-based models of care to address patient and family needs during this early survivorship period. The EMERGE model of care has been developed to provide eHealth-delivered, multidisciplinary care to patients and families in the 12 months following treatment for acute lymphoblastic leukemia, the most common type of pediatric cancer.

**Objective:**

The primary aim of this study is to assess the implementation success of the EMERGE model of care into the clinical setting. Secondary aims include evaluating effectiveness and cost consequences.

**Methods:**

The study uses a nonrandomized hybrid implementation-effectiveness design, assessing both implementation and clinical outcomes. Implementation metrics include evaluating the reach, acceptability, feasibility, and maintenance of the EMERGE model. Clinical effectiveness outcomes include parent satisfaction with the EMERGE intervention and pre-post intervention evaluation of parent psychological stress and unmet information needs. The Reach, Effectiveness, Adoption, Implementation, and Maintenance implementation science framework was used to guide study outcomes. Semistructured interviews with clinicians and parents will further evaluate the acceptability and sustainability of the EMERGE model and appraise barriers and facilitators to implementation. Cost analysis will include evaluation of the resources required for program delivery and the impact on subsequent health care service use measured using Medicare data and health service usage collected during the EMERGE intervention.

**Results:**

The trial commenced in December 2022, and recruitment concluded in October 2025, with 81 families recruited. Data collection is ongoing and is anticipated to be completed in Summer 2026.

**Conclusion:**

This study will address a critical gap in multidisciplinary care delivery at the end of treatment for young survivors of acute lymphoblastic leukemia and their families. The EMERGE model has the potential to improve the quality of life of patients and families by providing an early survivorship intervention. Importantly, the usage of an eHealth (telehealth) model will enable distance-delivered care, facilitating family participation regardless of geography. By measuring implementation, clinical, and cost impacts, this study will inform the future development of end-of-treatment models of care that are almost universally lacking in pediatric oncology care.

## Introduction

There is a critical gap in care for young cancer survivors after they complete their primary treatment [1,2]. Typically, young patients and their families transition from a multidisciplinary team of clinicians during active treatment to a model of medical surveillance in the off-treatment phase, with a period of at least 2 years and up to 5 years before they are eligible for “survivorship” programs. This gap in multidisciplinary clinical care represents a crucial missed opportunity to identify those children and adolescents at highest risk of educational, neurocognitive, psychosocial, and physical late effects from their cancer treatment and to implement preventative, targeted referrals and interventions early [2].

The end-of-treatment period is recognized as a potentially difficult transition for patients and families [2-6]. Children and adolescents have reported reduced physical functioning, lowered mood, and difficulties with anxiety, sleep, school re-entry, and social relationships [7-10]. Parents may experience elevated distress, loneliness, and fatigue [3,11,12], feelings of “abandonment” from their health care team [13-15], and an increased sense of personal responsibility, particularly in monitoring their child for relapse or adjustment difficulties [3,16]. Unmet information needs are high during the end-of-treatment period for both parents and young survivors, predominantly related to a lack of understanding of late effects and access to medical and psychosocial information [17-19].

The most common form of childhood cancer is acute lymphoblastic leukemia (ALL). Despite high survival outcomes [20], a substantial proportion of children and adolescents treated for ALL under modern chemotherapy-only regimens experience treatment late effects in the survivorship period (>5 y post treatment). Research indicates up to 50% of survivors will experience cognitive deficits in speed of information processing, working memory, attention, executive functions, and fine motor control [15,21], and behavioral and emotional symptoms including social withdrawal, anxiety, depression, somatization, inattention, and hyperactivity [22,23]. Pediatric ALL treatment also has a significant impact on parents and families more broadly, with disruption to daily routines and nearly every aspect of family life during active treatment [5,24,25]. While many parents and families will adapt over time, some parents experience persistent distress [26,27], and siblings are at risk for psychological distress, increased school absenteeism, and poorer academic outcomes [28,29].

Despite substantial evidence of the difficulties families face at the end of treatment, very little research has examined the provision of care during this period, particularly in relation to addressing the developmental and psychosocial needs of patients and families [9,15]. Therefore, it is currently not clear what is best practice in terms of the timing and mode of delivery of interventions [15,30]. However, it is highly feasible that intervening in this period may prevent the development of long-term deficits and enhance patients’ educational, social, and emotional outcomes [2,10,31,32]. It is also feasible that parents may be more able to address their own psychological needs and those of siblings once their child’s treatment has finished [6,15,33]. This juncture represents a crucial opportunity to intervene and optimize the recovery trajectory for these young survivors and their families.

In recognition of these difficulties, our research team has developed a novel, telehealth-delivered end-of-treatment model of care called the EMERGE program. EMERGE is designed to be delivered, via an advanced practice nurse and psychosocial clinician, for pediatric and adolescent patients who have completed treatment for ALL (or lymphoblastic lymphoma) within the last 12 months. Participants (parent or caregiver and child) receive 2 telehealth consultations from home and are provided with documentation, including a treatment summary, surveillance roadmap, letters summarizing consultations, and information resources. The EMERGE model was co-designed by an expert research team together with consumers (parents and adolescents) and oncology health care providers. If successful, the EMERGE model is designed to address unmet information needs, provide multidisciplinary clinical assessment of patient and family needs, and provide a preventative and early intervention approach to addressing physical, psychological, and developmental impacts of cancer therapy, thereby potentially reducing the long-term burden of cancer treatment late effects.

This study will address the current gap in systematized, evidence-based end-of-treatment interventions for young pediatric cancer survivors and their families, addressing both the implementation and effectiveness outcomes assessed across 2 pediatric cancer centers.

Within this paper, we present the approach to the design and investigation of the EMERGE program.

## Methods

### Study Aims

The primary aim of this study is to assess the implementation of the EMERGE model of care in 2 Australian pediatric cancer centers across a range of indicators, including reach, acceptability, satisfaction, maintenance, and barriers and facilitators to participation.

The secondary aim of the study is to evaluate the clinical effectiveness of the EMERGE model of care, including (1) participant satisfaction, (2) reduction in unmet information needs, and (3) reduction in parent or caregiver stress.

An exploratory aim of this study is to evaluate the costs of providing EMERGE and explore a sustainable model to upscale the program to all patients and families diagnosed and treated for childhood cancer in Victoria, Australia.

### Setting

This study will be conducted at the Children’s Cancer Centers at The Royal Children’s Hospital (RCH; parent site) and the Monash Children’s Hospital (MCH; satellite site) in Melbourne, Australia. These 2 centers are responsible for coordinating the care of all children and young people aged 0‐18 years diagnosed with cancer in the state of Victoria, Australia.

### Outcomes

#### Implementation Outcomes

We hypothesize that EMERGE will be successfully implemented at both study sites; specifically, the telehealth platform will be highly acceptable to participants, and stakeholders will report high levels of satisfaction with the intervention. We hypothesize that the barriers and facilitators to implementation will differ between the parent site (RCH) and satellite site (MCH) due to available resources and local champions. We also hypothesize that the intervention will be relatively low-cost to deliver, supporting the development of a sustainable business model for ongoing service provision once the research program ceases.

#### Clinical Effectiveness Outcomes

We hypothesize that the EMERGE program will (1) be rated highly (satisfaction) by participants, (2) decrease unmet information needs for participants, and (3) lead to a reduction in parent-perceived stress.

### Trial Design

This implementation study will use a mixed methods approach to develop and evaluate the EMERGE end-of-treatment model of care. A type 2 nonrandomized hybrid trial will be used. A hybrid design advances knowledge of both the intervention and the implementation factors associated with integration into clinical practice [34].

This study has a dual focus of assessing (1) the implementation outcomes of the EMERGE model of care in real-world settings across 2 institutional sites, and (2) the clinical effectiveness of the EMERGE model of care using a within-subject pre-post design.

The study is guided by the Reach, Effectiveness, Adoption, Implementation, and Maintenance (RE-AIM) framework [35]. RE-AIM emphasizes the translation of research into practice and the importance of considering context in intervention planning and design. It provides a framework to assess what intervention components are effective, for whom and in which settings, and with which implementation strategies [36]. RE-AIM theorizes that the impact of an intervention is a function of 5 systems based on socioecological factors, that is, reach, efficacy, adoption, implementation, and maintenance. The proposed evaluation embedded within this protocol addresses these 5 factors.

### Ethical Considerations

Before study activation and participant enrollment, the study protocol was approved by the RCH Human Ethics and Research Committee (78840). Informed consent is obtained from all participants aged 16 years and older. Parental consent will be obtained for children younger than 18 years of age. All data are securely stored on servers and software approved for the use of protected health information. Additionally, data are deidentified upon collection and stored under a study identification number. Participants did not receive compensation for participation in this trial.

### Participants

Children and adolescents aged between 2 and 18 years (or older if receiving active surveillance in the pediatric setting), who are completing treatment for pediatric ALL at 1 of the 2 cancer centers (RCH and MCH), and a parent caregiver will be invited to participate in the study. Patients who have completed treatment for lymphoblastic lymphoma are also eligible for this study, as they present with the same disease features and undergo the same treatment protocols as patients with ALL. Given the high similarities shared by these disease entities, and for ease of reference, we will refer to patients with ALL in this protocol. Refer to Table 1 for participant criteria.

**Table 1.** Eligibility criteria.

	Inclusion criteria	Exclusion criteria
Patients	Child aged 2‐18 y (or older if receiving active surveillance in the pediatric setting). Diagnosed with ALL^a^ or LLy^b^ Treated at the RCH^c^ or MCH^d^ Is within the last 3 months of maintenance therapy or has completed treatment within 12 months at time of recruitment.	Outside this age range Diagnosed with another form of cancer With relapsed or refractory disease Receiving end-of-life treatment
Parent or caregivers	Parent of a patient aged between 2 and 18 y (or older if receiving active surveillance in the pediatric setting), who has been treated for ALL or LLy at either the RCH or MCH and is in the final period maintenance treatment or has completed treatment within the last 12 months.	Parent of a child with another form of cancer, or who has relapsed or refractory disease and/or is receiving end-of-life treatment.
Health care providers	Medical, nursing, social work, allied health, and mental health staff, heads of departments and champions, who work with children treated for ALL or LLy and their families at either the RCH or MCH.	Medical, nursing, social work, allied health, or mental health staff who do not work directly with pediatric patients with ALL or LLy.
Institutional champions	Head of Department, Operational Managers, Executive staff	—^e^

a ALL: acute lymphoblastic leukemia.

b LLy: lymphoblastic lymphoma.

c RCH: Royal Children’s Hospital.

d MCH: Monash Children’s Hospital.

e Not applicable.

### Recruitment

Children and adolescents with ALL, being treated with curative intent (in the nonrelapse or refractory setting) and who have recently completed treatment or are nearing the end of treatment will be identified during routine multidisciplinary team meetings undertaken weekly at the RCH and MCH cancer centers. Families identified as potential participants will be introduced to the program by their treating oncologist and provided with a study brochure. For families who do not opt out of the study, a member of the research team will contact the primary caregiver directly to obtain written consent to participate in the study. Adolescents will be approached to provide consent if they are determined to have appropriate literacy ability and cognitive capacity to understand the requirements of the intervention. Competency will be determined by talking to the treating team and in consultation with parents and caregivers. For culturally and linguistically diverse families, interpreter services can be accessed to assist with the introduction and enrollment of families into the study. We will also encourage families to include a support person in this process if they find this helpful.

Health care providers and organizational stakeholders will be approached directly by the research team to participate in the project. For data that are collected directly from study participants (eg, questionnaires), the participant will need to provide electronic consent via REDCap (Research Electronic Data Capture; Vanderbilt University). REDCap is a secure, web-based software platform designed to support data capture for research studies [37,38].

### The EMERGE Intervention Model

The EMERGE model of care will offer families a minimum of 2 telehealth (video conference) consultations during the first 12 months of their child completing treatment for ALL. The EMERGE program is cofacilitated by an advanced practice nurse and psychosocial clinician who have been trained in the implementation of the intervention. A telehealth platform was chosen to address barriers identified for attending appointments (eg, burden of attending the hospital, desire to avoid health settings, and disruption to school attendance) and to increase the opportunity to deliver the intervention regardless of geographical location from the hospital. Telehealth has strong organizational support from the hospitals and offers a flexible, safe, and potentially cost-effective framework to screen and monitor families in the off-treatment period [39,40].

**Figure 1.**
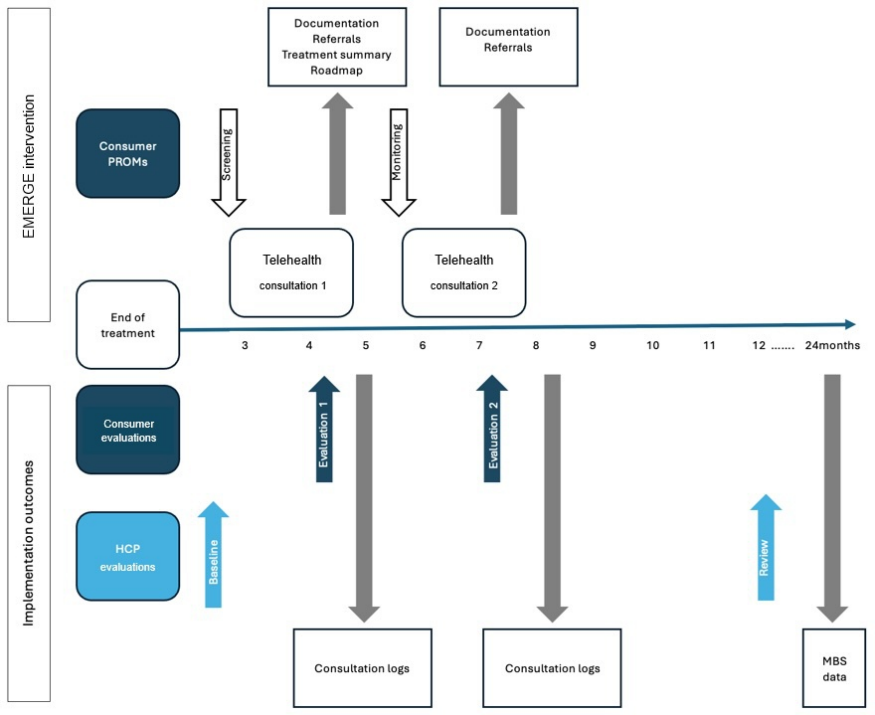
Schematic diagram of the EMERGE model of care. HCP: health care provider; MBS: Medicare Benefits Scheme; PROM: patient-reported outcome measure.

The EMERGE model is based upon the Pediatric Psychosocial Preventative Healthcare Model, a 3-tier model of universal, targeted, and clinical categorization that represents the distribution of psychosocial risk across family populations [41]. The EMERGE model also incorporates the recommendations of the Standards for the Psychosocial Care of Children with Cancer and Their Families, developed in 2015 [42], by specifically including components, such as psychosocial screening, psychoeducation, monitoring and review, and specialist interventions (Figure 1).

### Components of the EMERGE Model

The key components of the EMERGE model are presented below.

First, identification of eligible participants via the clinical team and informed consent obtained by the EMERGE research team.

Second, treatment summary and surveillance roadmap: these documents will be prepared by the EMERGE advanced practice nurse and validated by the patient’s primary oncologist. The treatment summary includes the child’s initial presentation, significant medical and family cancer history, the treatment protocol delivered, diagnostic criteria (including criteria for risk stratification), cumulative chemotherapy doses, significant treatment toxicities, and any clinical trial participation. The surveillance summary provides a schedule of the necessary tests, investigations, and physical examinations over the corresponding 5-year period. These documents provide a narrative for the nurse to revisit the diagnosis and treatment with the participants, as well as to discuss the prospective treatment and surveillance plan.

Third, screening: a risk profile is established for each patient or family based upon the treatment summary and administration of validated patient-reported outcome measures (PROMs). Screening PROMs include measures of patient symptom burden and quality of life (QoL), parent distress and QoL, and family psychosocial risk and functioning (refer to Table 2 for complete list). These measures are completed by the parent caregiver and patients aged >8 years at preintervention baseline (screening) to inform the first telehealth session, with selected measures repeated before the second telehealth session (monitoring).

**Table 2.** Screening measures for EMERGE consultations.

Intervention and health measures	Participant and data source	Timing and data collection	Measure
Psychosocial assessment			
Psychosocial risk	Parent	Screening^a^	Psychosocial Assessment Tool, Version 3 (Australian adaptation)
Patient assessment			
Stress	Parent proxy Child^b^	Screening Monitoring^c^	PROMIS^d^ Stress Experiences Scale (4-item)
Summary profile	Parent proxy Child^b^	Screening Monitoring	PROMIS summary profile (25-item): includes physical function (mobility), anxiety, depressive symptoms, fatigue, peer relationships, pain interference, and pain intensity.
Cognition	Parent proxy Child^b^	Screening Monitoring	PROMIS Cognitive function (7-item)
Physical function	Parent proxy Child^b^	Screening Monitoring	PROMIS Physical activity (4-item)
Sleep issues	Parent proxy Child^b^	Screening Monitoring	PROMIS Sleep-related disturbance (4-item) and PROMIS Sleep-related impairment (4-item)
Meaning and purpose	Parent proxy Child^b^	Screening Monitoring	PROMIS Meaning and Purpose (4-item)
Friendships	Parent proxy Child^b^	Screening Monitoring	NIH^e^ Toolbox Friendship (5-item)
Social relations	Parent proxy Child^b^	Screening Monitoring	Neuro-QOL^f^ Pediatric Social Relations – interactions with peers (8-item)
Parent assessment			
Stress	Parent	Screening Evaluation 1^g^ Evaluation 2^h^	NIH Toolbox perceived stress module (10 items)
Distress	Parent	Screening Monitoring	Distress Thermometer for Parents (single item plus 34 item problem list)
Family assessment			
Family functioning	Parent	Screening	McMaster Family Assessment Device (12 items)

a Screening: before consultation 1.

b Child aged more than 8 y.

c Monitoring: before consultation 2.

d PROMIS: Patient-reported Outcomes Measurement Information System.

e NIH: National Institutes of Health.

f Neuro-QOL: Quality of Life in Neurological Disorders.

g Evaluation 1: following consultation 1.

h Evaluation 2: following consultation 2.

Fourth, psychoeducation or resource provision: psychoeducation and counseling will be provided directly by the EMERGE advanced practice nurse and psychosocial clinician during the 2 scheduled telehealth sessions. Telehealth sessions will occur at least 3 months apart for families with a minimum of 2 consultations. Information resources will be provided both electronically and in hard copy if required and will directly address concerns identified in each of the EMERGE telehealth sessions (eg, immunization schedule, sleep, when to call the hospital, fatigue, financial resources, and cancer charities).

Fifth, documentation: a summary letter, personally addressed to the parent and, if applicable, adolescent, will be provided both electronically and in hard copy to families after each EMERGE consultation, along with the treatment summary, roadmap, and information resources. With parent permission, these documents will also be shared with the child’s general practitioner or pediatrician. Documents will also be sent to the primary oncologist and uploaded into the patient’s electronic medical record.

Sixth, monitoring and review: follow-up surveys after completion of the first EMERGE consultations will evaluate satisfaction with the consultation, parent stress, and information needs. PROMs are readministered before the second EMERGE consultation to enable monitoring of patient and family well-being. Goal or intervention adherence, health service usage, and referral needs will be monitored and discussed in the second EMERGE consultation. Families will be provided access to EMERGE clinicians via a dedicated email and telephone number, which will be monitored regularly by the research team.

Finally, specialist intervention: for patients and families with significant risk factors and identified concerns, referrals will be actioned to hospital or community-based specialists (eg, clinical psychology, consultant pediatrician, neuropsychology, occupational therapy, education liaison, and medical oncologist). Patients and families will also be able to escalate their concerns to activate an additional telehealth or face-to-face session with their EMERGE clinicians or specialist medical consultants as required.

### Measures

#### Screening Measures

Refer to Table 2 for a summary of screening measures and the administration schedule.

#### Validated Questionnaires Administered

##### Psychosocial Assessment Tool – Third Edition

The Psychosocial Assessment Tool – Third Edition (PAT 3) [43] is a brief parent report screener of family psychosocial risk. The PAT 3 generates a total score and 7 subscale scores (family structure, social support, child problems, sibling problems, family problems, stress reactions, and family beliefs) by summing the number of endorsed high-risk items. The total score has cut-offs for 3 tiers of risk—universal, targeted, and clinical. The PAT 3 has demonstrated good internal consistency (Kuder-Richardson coefficient=0.81) and construct validity [43].

##### Patient-Reported Outcomes Measurement Information System

The Patient-Reported Outcomes Measurement Information System [44] measures are a system of highly reliable and precise measures to evaluate patient-reported physical, mental, and social health. They are validated for paper or computer completion by parent proxy (age 5‐7 y) and by self-report (age >8 y). Measures assess a range of symptom domains, including physical and motor function, sleep, cognition, stress, relationships, physical activity, and life satisfaction. Internal consistency has been shown to be strong (Cronbach α=0.72-0.92) [45], as is construct validity [46,47] and temporal sensitivity [47] in pediatric oncology samples. This study will use the Patient-Reported Outcomes Measurement Information System summary profile (25 items) with additional domains included (eg, cognition, sleep impairment, meaning, and purpose).

##### Distress Thermometer for Parents

The Distress Thermometer for Parents [48] consists of 3 sections examining parent distress levels and specific problems over the previous week. Parents indicate their distress on a thermometer scale ranging from 0 (*no distress*) to 10 (*extreme distress*); a score of 4 or higher is indicative of clinically elevated distress. They also complete a 34-item problem list spanning 6 domains: practical, social, emotional, physical, cognitive, and parenting. Finally, questions regarding perceived support and understanding from others, parental chronic disease, relationships with medical staff, and desire to consult a health professional about the situation are completed. The Distress Thermometer for Parents has been validated as a short screening tool for identifying parental distress [48].

##### McMaster Family Assessment Device

The McMaster Family Assessment Device [49] is based on the McMaster model of family functioning and contains statements concerning the family’s functioning. The measure requires respondents to indicate their level of agreement with each statement on a 4-point Likert scale *(*strongly agree to strongly disagree). Higher scores indicate poorer family functioning. For this study, only the General Family Functioning Scale will be used (12 items), and it has been shown to have good reliability and validity [50].

### Implementation Measures

Table 3 summarizes the implementation measures mapped according to the RE-AIM framework. These measures evaluate implementation processes and any factors (barriers and enablers) influencing implementation of the EMERGE model of care.

**Table 3.** Implementation measures using the RE-AIM^a^ framework.

Implementation outcome and tool	Source	Phase
Reach		
Percentage uptake among eligible families		
Recruitment log	Trial database	End of trial
Representativeness of participants for Victorian ALL^b^ population		
Recruitment log	Trial database	End of trial
Acceptability of model		
Experience of Care survey	Consumers	Evaluation 1^c^ Evaluation 2^d^
Semistructured interviews (subset)	Consumers HCPs^e^	Evaluation 2
Acceptability of the telehealth platform		
Experience of care survey	Consumers	Evaluation 1 Evaluation 2
Semistructured interviews (subset)	Consumers HCPs	Evaluation 2 12 months
Effectiveness		
Information needs		
Information Needs survey	Consumers	Screening Evaluation 1 Evaluation 2
Satisfaction with intervention		
Client Satisfaction Questionnaire	Consumers	Evaluation 1 Evaluation 2
Experience of care		
Experience of Care survey	Consumers	Evaluation 1 Evaluation 2
Semistructured interview (subset)	Consumers	Evaluation 2
Health service usage		
Health services usage log	Consumers	Evaluation 2
Adoption		
Participation rates		
Recruitment log	Trial database	End of trial
Number of staff trained in EMERGE administration		
Training Log	Trial database	End of trial
Implementation		
Percent of survivorship care plans provided		
Care plan log	Trial database	End of trial
Adherence		
Referral log	Trial database	End of trial
Recommendations log	Trial database	End of trial
Percent of participants who completed 2 telehealth sessions		
Telehealth session log	Trial database	End of trial
Percent of participants who received an information resources pack		
Resource allocation log	Trial database	End of trial
Acceptability, appropriateness, and feasibility of the intervention		
Acceptability Intervention Measure, Intervention Appropriateness Measure, and Feasibility of the Intervention Measure	HCPs	Baseline^f^ 12‐24 months
Maintenance		
Staff undertaking procedures		
Telehealth session log	Trial database	Evaluation 1 Evaluation 2
Follow-up work log	Trial database	Evaluation 1 Evaluation 2
Length of consultation		
Telehealth session log	Trial database	Evaluation 1 Evaluation 2
Time spent on follow-up		
Follow-up work log	Trial database	Evaluation 1 Evaluation 2
Sustainability of EMERGE model		
Health Economics Evaluation		
CHU9D^g^ and AQoL-4D^h^	Consumers	Screening^i^ Evaluation 2 12 months
MBS^j^ and PBS^k^ data	Services Australia	24 months
Cost of model integration into service	Trial database	Evaluation 2
HCP and institutional evaluation		
Semistructured interviews	HCPs	12‐24 months
Semistructured interviews	Executive staff	End of trial

a RE-AIM: Reach, Effectiveness, Adoption, Implementation, and Maintenance.

b ALL: acute lymphoblastic leukemia.

c Evaluation 1: following consultation 1.

d Evaluation 2: following consultation 2.

e HCP: health care provider.

f Baseline: beginning of trial implementation.

g CHU9D: Child Health Utility 9D.

h AQoL-4D: Australian Quality of Life Instrument.

i Screening: before consultation 1.

j MBS: Medicare Benefits Scheme.

k PBS: Pharmaceutical Benefits Scheme.

#### Acceptability of Intervention Measure, Intervention Appropriateness Measure, and Feasibility of Intervention Measure

The Acceptability of Intervention Measure, Intervention Appropriateness Measure, and Feasibility of Intervention Measure [51] are 3 implementation measures designed to measure the degree of fit or match of something to a desired criterion and allow for evaluating the success of the implementation efforts. These measures specifically assess the acceptability, appropriateness, and feasibility of the intervention. Responses are made on a 5-point ordinal scale ranging from “*completely disagree*” to “completely agree.” Each of the measures has been shown to have good reliability (Acceptability of Intervention Measure Cronbach α=0.85, Intervention Appropriateness Measure Cronbach α=0.89, and Feasibility of Intervention Measure Cronbach α=0.89) as well as good conceptual validity [51]. These measures will be completed by the health care provider before and after the implementation of the EMERGE intervention.

#### Semistructured Interviews

A subset of parents (n=20, 25%) and health care providers (n=10‐15) from the RCH and (n=5‐10) from MCH will be asked to complete a semistructured interview (with a researcher not involved in delivering the program), 12‐24 months following the EMERGE intervention being active at their site. Interviews will include questions related to the acceptability of the EMERGE model, experience of care, barriers and facilitators to the delivery, and sustainability of the EMERGE model.

### Outcome Measures

#### Information Needs Survey

An information needs survey has been developed for this study and was adapted from an original survey used in the Adolescent and Young Adult Health Outcomes and Patient Experience (AYA Hope) study [52] and a subsequent Australian study of adolescent and young adult cancer survivors [53]. Parent participants and adolescent participants older than 16 years will be asked to rate their need for more information on items such as their treatment side effects, cancer relapse, physical functioning, survivorship plan, and community-based services. Participants respond to each of the 31 items, indicating (1) *I have enough information*, (2) *I need some more information*, (3) *I need much more information*, and (4) *does not apply*.

#### National Institutes of Health Toolbox Perceived Stress Scale

The National Institutes of Health Toolbox Perceived Stress Scale [54] is a self-report measure of an individual’s perceptions about the nature of events and their relationship to the values and coping resources of an individual. This is a 10-item measure, rated on a 5-point Likert scale (0*=never* to 4*=very often*). It assesses how unpredictable, uncontrollable, and overloaded respondents find their lives. The perceived stress module is normed from 18 to 85 years of age and provides a *t* score with a mean of 50 (SD 10). The Perceived Stress Scale has demonstrated good reliability (Cronbach α=0.91) and content validity [54].

#### The Client Satisfaction Questionnaire

The Client Satisfaction Questionnaire [55] is a self-report measure of satisfaction with services received by individuals and families in the health and human services settings. The measure has 8 items that target: quality of service, kind of service, met needs, recommend to a friend, amount of help, deal with problems, overall satisfaction, and return intent. Responses are made on a 4-point Likert scale, with high scores indicating higher satisfaction. Reliability and validity testing in health care contexts support good reliability (Cronbach α=0.83-0.93) and validity [55].

#### Experience of Care Survey

This survey has been developed specifically for the EMERGE study and adapted from other health intervention implementation studies. The survey comprises 7 questions which parents respond to on a 5-point Likert-type scale ranging from strongly agree to strongly disagree. Example items include “*I was able to ask all the questions I had about my child*,” “*There was enough time to cover all the areas I wanted to discuss during the session*,” and “*The telehealth platform was easy to use*.”

### Health Service Usage

#### Medicare Benefits Schedule Data and Psychosocial Service Usage

To capture health service usage during the period of intervention, we will obtain consent (optional) to access the patient’s Medicare Benefits Schedule data via Services Australia. This consent will be sought by the research team to align with the family’s second telehealth session. The data will provide information relating to Medicare Benefits Schedule–billed medical visits and procedures, and the associated costs (eg, general practitioners, diagnostics, and specialists, including psychosocial services where Medicare is subsidized). Data will be requested for a 24-month period post-treatment.

##### Child Health Utility 9D

The Child Health Utility 9D (CHU9D) [56] is a preference-based measure of health-related QoL for children and adolescents. Parent-proxy forms will also be used to monitor patient QoL in this study. The questionnaire assesses 9 dimensions—worries, sadness, pain, tiredness, annoyance, schoolwork, sleep, daily routine, and activities. Responses are made on a 5-point ordinal scale of “*no*” to “very.” The CHU9D consists of a descriptive system and a set of preference weights, giving utility values for each health state described by the descriptive system, allowing the calculation of quality adjusted life years (QALYs). The measure has been shown to have good reliability [57] and construct validity [58].

##### The Australian Quality of Life Instrument

The Australian Quality of Life Instrument (AQoL-4D) [59] is a multiattribute utility health-related QoL instrument, which will be used to monitor parent QoL in this study. The AQoL-4D consists of 12 items assessing 5 dimensions: illness, independent living, social relationships, physical senses, and psychological well-being. Each item has 4 response levels. The AQoL-4D uses a descriptive system and set of preference weights that provide utility values for each health state described by the descriptive system. As with the CHU9D, this will allow for the calculation of QALYs [59].

### Data Analysis

#### Sample Size Estimation

The RCH and MCH collectively treat approximately 90 children and adolescents annually who are diagnosed with ALL. Given that approximately 15% (n=9) of these patients may relapse, become palliative, or die, and recruitment will occur over a 24 to 30-month period, we anticipate recruitment of approximately 100 families over the course of the project (allowing for some families opting not to participate and some withdrawals due to disease recurrence).

#### Data Analysis Plan

Feasibility and acceptability of the EMERGE model will be assessed by calculating recruitment and participation rates, acceptability ratings by clinical staff, and parents’ or patients’ satisfaction measured post-intervention (eg, length, content, and information). Descriptive statistics will be used to report sample characteristics and for all quantitative measures. Intention to treat will be used to analyze primary outcome measures (satisfaction, information needs, and parent stress ratings). Change between time points in information needs and stress ratings will be analyzed using paired *t* tests. In order to detect a clinically meaningful change from baseline of 0.3 of an SD, with 80% power and error rate α=.05, a sample size of n=100 is required. Univariate and multivariate analyses will be used to identify psychosocial, demographic, and treatment-related variables associated with parent information needs and stress ratings. With a sample of n=100 (80% power, α=.05), we will be powered to detect a medium size of effect (*f*^2^=0.15) in multiple linear regression models with up to 5 predictors. Secondary outcomes such as uptake or adherence to recommendations will be calculated (frequencies and percentages).

#### Costs and Consequences

The assessment of costs will reflect the resources required for program delivery (as reflected by the type of practitioner involved in the delivery of the program and the time required) and the impact on subsequent health care service use (as reported from Medicare data and patient information for psychology services). Costs will be reported on the basis of total and average costs per patient for program delivery, subsequent health care use, and overall. Program consequences will be expressed in terms of the impact from baseline on unmet needs, satisfaction, and QALYs (as separate outcomes).

#### Qualitative Analysis

Health care provider and parent interviews will be analyzed deductively to code a priori themes related to implementation domains. Inductive analysis will also be used to identify novel or emerging data, using the thematic approach described by Braun and Clarke [60]. Deidentified interview transcripts will be imported into Lumivero NVivo software [61] for coding and analysis. Parent and health care provider interviews will be analyzed separately. Data analysis will examine differences between stakeholders and participants and across sites and will examine barriers and facilitators to the delivery of the EMERGE intervention.

## Results

Funding for this study was received from the Victorian Cancer Agency in April 2021. The trial commenced in December 2022, and recruitment concluded in October 2025, with 81 families recruited. Data collection is ongoing and is anticipated to be completed in Summer 2026. Data analysis and results are expected to be completed in late 2026.

## Discussion

### Principal Findings

Consistent data from many research studies highlight that the end-of-treatment period is a difficult transition period for many patients and families [1,2]. Furthermore, survivors of childhood cancer are often impacted by disease and treatment-related late effects, but few interventions have been developed that adopt a preventative approach to identifying issues and proactively providing strategies and resources to optimize child and family well-being [6,62,63]. This protocol details a type 2 nonrandomized hybrid implementation-effectiveness trial that will evaluate an eHealth, multidisciplinary end-of-treatment model of care for pediatric patients who have completed treatment for ALL and their families. The EMERGE intervention addresses a critical gap in models of care at this very early survivorship period and bridges the gap between active treatment and later survivorship models.

The study will assess implementation outcomes using both quantitative and qualitative measures that will provide a detailed assessment of the feasibility and acceptability of the EMERGE model as well as barriers and facilitators. The hybrid methodology, examining implementation and effectiveness outcomes, will provide important data to guide future research and clinical efforts, given the current dearth of end-of-treatment interventions. Health economic analyses will also ascertain the relative costs of the program and contribute to efforts to achieve sustainability of such models of care into the clinical setting.

### Strengths and Limitations of This Study

A significant strength of the EMERGE model is that it is delivered by multidisciplinary staff (advanced practice nurse and psychosocial clinician) who are able to respond to medical and psychosocial issues in real time and with the requisite expertise. This is an important feature of the EMERGE model, given the predominance of psychosocial or mental health issues reported by families during this period [3,64]. A further strength of the study is the focus on implementation outcomes, which, given the small number of published evidence-based models of care, will significantly contribute to understanding the barriers and facilitators to establishing end-of-treatment programs. The delivery of the program across 2 hospital sites will provide additional information related to contextual and organizational factors that may influence implementation. Delivery of the program via telehealth will enhance the accessibility of the program to families [39]. This is an important consideration, particularly in the Australian context, where the population is geographically dispersed and traveling to the hospital for additional visits is costly and difficult for many families [65]. We anticipate that the use of telehealth, and also additional resources to support the participation of culturally and linguistically diverse families, will potentially increase the equity and accessibility of the program for all families, and essential considerations in designing any new model of clinical care.

Limitations of the study must be noted. As the study is focused only on patients with ALL and their families, findings will not necessarily be able to translate to other diagnoses. Furthermore, despite the convenience of the telehealth model, this may also be less optimal in terms of engaging young children and adolescents, and some families may indicate a preference for in-person consultations. Finally, the nonrandomized study design means the clinical effectiveness outcomes should be interpreted with caution. The within-subject, pre-post design for clinical measures (parents’ stress and information needs) cannot exclude natural recovery or regression to the mean. Future study design considerations should consider ethical and innovative study designs, including randomized controlled designs, to measure whether interventions such as EMERGE prevent or impact short- and longer-term survivorship outcomes for young people and their families.

### Conclusion

Addressing the complex needs of a growing population of pediatric cancer survivors is a challenge for health care systems. The EMERGE intervention offers a potentially effective and low-cost approach to addressing the unmet informational, clinical, and psychosocial needs of young survivors of ALL and their families. Importantly, the model also fosters families’ re-engagement with primary care providers (eg, general practitioners), which is an important factor in optimizing patient and family well-being as hospital resources are reduced following active treatment. The focus of this study on implementation barriers and facilitators and cost analysis will provide important information to oncology staff and hospital administrators who must continue to promote the evolution of health care models to address the significant treatment-related morbidity associated with increased pediatric cancer survivorship.

## Supplementary material

10.2196/85901 Peer Review Report 1 Peer review report by The Royal Children's Hospital Melbourne and the Australian and New Zealand Children's Haematology and Oncology Group.

## Abbreviations

ALL: acute lymphoblastic leukemia
AqoL-4D: Australian Quality of Life Instrument
CHU9D: Child Health Utility 9D
MCH: Monash Children’s Hospital
PAT 3: Psychosocial Assessment Tool – Third Edition
PROM: patient-reported outcome measure
QALY: quality adjusted life year
QoL: quality of life
RCH: Royal Children’s Hospital
RE-AIM: Reach, Effectiveness, Adoption, Implementation, and Maintenance
REDCap: Research Electronic Data Capture
