# Supplementary material for: Implementing an eHealth Model of Care for Pediatric Patients and Families at the End of Treatment for Acute Lymphoblastic Leukemia (EMERGE): Type 2 Nonrandomized Hybrid Implementation-Effectiveness Trial Study Protocol
Source: JMIR Res Protoc. 2026 Feb 26;15:e85901. doi: 10.2196/85901 (PMC12945096; doi:10.2196/85901)
Supplement: Peer Review Report 1 [file resprot-v15-e85901-s001.pdf]

The Royal Children's Hospital Melbourne

Pre-Submission (Peer) Review Proforma

The purpose of the review is to identify areas for improvement which will ensure the project is scientifically valid. Please also see Pre-submission (Peer) Reviewer Process for more information.

**PROJECT TITLE**	An EHealth Survivorship Model of Care at the End of Treatment for Survivors of Child and Adolescent Acute Lymphoblastic Leukaemia and their families: The **Emerge** study
**Version number & date of Protocol under review**	Version I, 12th January 2022

**PRINCIPAL INVESTIGATOR**	A/Prof Maria McCarthy
**Position Title**	Team Leader, Cancer and Chronic Illness
**Department / Group**	Clinical Sciences/Brain and Mind
**Institution**	Murdoch Children's Research Institute

**PEER REVIEWER NAME**	Prof Stephanie Best
**Position Title**	Honorary Fellow Manager
**Department / Group**	Genomics Health Alliance
**Institution**	Murdoch Children's Research Institute

PEER REVIEWER DECLARATION:

- I agree to maintain confidentiality of all matters and documents regarding this project; and
- I am independent of this project; and

☒ I agree that I have no potential conflicts of interest in reviewing this research protocol; **OR**

☐ I declare I have the following potential conflicts of interest:

Please disclose any actual or potential conflict of interest in the research being reviewed, including any:

a) Personal involvement or participation in the research
b) Financial or other interest or affiliation, or

c) *Involvement in competing research*

Please explain any 'No' response and also record any comments regarding required changes or suggestions which could improve the project in the relevant section on the last page of this form.

**CRITERIA:** *Using the right column please indicate if each criteria has been addressed, in your opinion*		**YES NO N/A**
**Project details:** Has all appropriate information been included? (Investigator details and project title, protocol version number and date)		yes
**Research question:** Is there a clearly and precisely defined, answerable question? Is there a clear aim or objective?		yes
**Background:** Is the research question an important one? Does the background information provided give a good rationale for why the project is being done? Is the study useful to clinical practice? Is there a real problem/ knowledge gap that needs filling?		yes
**Plan of Investigation:**		
1	**Design:** is the design appropriate to the aim? Will the study address the question being asked and is it likely to produce an answer?	yes
2	**Bias and confounding**: Has the study been designed to minimise the risk of bias? Have the investigators adequately accounted for the influence of potential confounders?	na
3	**Randomisation and Blinding:** Where applicable, is enough detail provided on exactly how randomisation and blinding will be achieved, including who is responsible?	na
4	**Sampling issues:** Will the proposed study group be large enough to provide sufficient statistical precision or power, where appropriate? Is there a reasonable justification for the proposed sample size? Will the sample collected be reasonably representative of the population in question?	na
5	**Feasibility:** Is there sufficient evidence to indicate that it will be possible to obtain the numbers required for the study? Is the study feasible in terms of funds, time and other resources?	yes
6	**Participants:** Are the criteria for eligibility clear and justified? Have the methods used to identify, approach, recruit and consent participants been clearly and completely described?	yes
7	**Intervention or exposure:** Is the intervention or exposure factor clearly described in adequate detail, where appropriate? If the intervention is a drug, are details of dose, delivery, preparation, handling and compliance provided?	yes
8	**Procedure plan:** Has an appropriate plan of the study been detailed? Is the estimated duration of the project stated and appropriate? Is it clear how a participant will progress through treatments, procedures, assessments and visits, where applicable?	yes
9	**Outcome measures:** Are these appropriate and achievable? Are definitions sufficiently detailed? Is the relevant data being collected on the proposed outcomes?	yes
10	**Adverse Events:** Is there an appropriate plan for detecting, managing, recording and reporting defined adverse events, where applicable?	yes

11	**Data collection:** are the proposed data collection tools and data management systems appropriate for the project?	yes
12	**Analysis:** is there an adequate indication of what analysis will be done on outcome measures to answer the research question? Are the proposed analyses appropriate?	yes
**Project management:** have adequate arrangements been specified for conduct and oversight?		yes
**Expertise:** Does the research team include (or have access to) all the necessary expertise for the project?		yes
**Ethical issues:** Have any potential ethical issues been addressed? Are risks to participants minimised?		yes

Each question, comment, suggestion or requirement should be separately bulleted.
Where applicable please reference the section and page number.

**General Comments** (Remarks that the investigator does not need to respond to)
▪ **This is an excellent study to investigate a much needed service for patients and families with ALL. The findings from this study will provide a valuable contribution to the literature and service delivery.**
**Required Changes** (Points that the investigator must address by either making the required change, or producing a cogent argument against the change)
▪ **At times the staff data collection is omit e.g. when discussing consent.**
**Suggested Changes** (Points that the reviewer thinks may improve the project. They are not of such importance that they would render the project scientifically invalid/unethical if the investigator did not address the issues)
▪ **I've made a few suggestions in the text that I hope may help improve the study. Please see attached.**

**Pre-submission review outcome:** (reviewer to circle)	
**A**	**No changes required:** take the study forward to submission.
**B**	**Changes suggested:** at the discretion of the investigator; take the study forward to submission.
**C**	**Changes required:** decision about acceptability of subsequent changes at the discretion of the HREC representative. A peer reviewer does not need to review the amended protocol prior to submission.
**D**	**Changes and further peer-review required:** decision about acceptability of the subsequent changes at the discretion of the reviewer following a pre-submission peer review of the amended

	protocol. An additional review & proforma should be completed to document the review of the amended protocol.

**Peer Reviewer Signature**		**Date**	18.1.22
